# Inhibitory Effects and Mechanisms of Perilla Essential Oil and Perillaldehyde against Chestnut Pathogen *Botryosphaeria dothidea*

**DOI:** 10.3390/jof10080526

**Published:** 2024-07-28

**Authors:** Qi Zeng, Lu Wang, Sha Long, Wanrong Dong, Yaoyao Li, Yuxin Chen, Gao Zhou

**Affiliations:** 1 Hubei Key Laboratory of Industrial Microbiology, Key Laboratory of Fermentation Engineering (Ministry of Education), Cooperative Innovation Center of Industrial Fermentation (Ministry of Education & Hubei Province), Hubei University of Technology, Wuhan 430068, China; zqi99@foxmail.com (Q.Z.); wanglu611@foxmail.com (L.W.); longshahbut@foxmail.com (S.L.); dongwwwrr@163.com (W.D.); yyl030425@163.com (Y.L.); yuxinc@hbut.edu.cn (Y.C.); 2 National “111” Center for Cellular Regulation and Molecular Pharmaceutics, School of Life and Health Sciences, Hubei University of Technology, Wuhan 430068, China; 3 Post-Doctoral Research Center of Mayinglong Pharmaceutical Group Co., Ltd., Wuhan 430064, China

**Keywords:** perilla, perillaldehyde, essential oil, *Botryosphaeria dothidea*, antifungal mechanism

## Abstract

*Botryosphaeria dothidea*, a notorious plant pathogen, is responsible for causing chestnut rot during postharvest storage. This research aimed to assess the antifungal properties of perilla essential oil (PEO) and perillaldehyde (PAE) against *B. dothidea*. PEO’s and PAE’s inhibitory effects on *B. dothidea* were investigated using an agar dilution method, a fumigation method, and an in vivo assay in chestnuts and shell buckets. Based on the results of gas chromatography-mass spectrometry, it was confirmed that the main component of PEO was elemicin. The antifungal mechanism of PEO and PAE against *B. dothidea* was investigated by conducting staining experiments of the fungal cell wall and cell membrane. PEO and PAE strongly inhibit the mycelial growth of *B. dothidea* in a dose-dependent manner. The inhibitory mechanism is mainly related to the destruction of the integrity of the fungal cell wall and plasma membrane. Notably, PEO retains its antifungal efficacy against *B. dothidea* in chestnuts, effectively prolonging their storage life. These findings indicate that PEO and PAE are nontoxic, eco-friendly botanical fungicides, holding promise for controlling postharvest chestnut rot.

## 1. Introduction

Chestnuts (*Castanea* spp.) are a distinctive group within the Fagaceae family, comprising several economically and ecologically significant tree species. The principal members of the *Castanea* genus cultivated worldwide are the Chinese chestnut (*Castanea mollissima*), Japanese chestnut (*Castanea crenata*), American chestnut (*Castanea dentata*), and European chestnut (*Castanea sativa*). Of these members, the Chinese chestnut boasts a rich legacy of cultivation, occupying a dominant position in global production [1]. Chestnuts are highly nutritious, boasting a substantial content of starch, fiber, amino acids, and vitamins, which significantly contribute to their dietary worth [2]. In addition to their nutritional value, chestnuts continue to have a wide range of applications. Lee et al. [3] demonstrated the anti-obesity effects of enzymatically modified chestnut starch in a diet-induced obese mouse model. According to Sangiovanni et al. [4], proanthocyanidins obtained from chestnuts have the potential to develop nutraceuticals with anti-gastritis properties. Sverguzova et al. [5] determined through chemical modification of chestnut shells that their sorption capabilities could be enhanced, thereby improving the removal of heavy metal ions and effectively boosting their pollutant adsorption properties.

The culinary versatility and wide-ranging applications of chestnuts have yet to be translated into proportional economic gains, primarily due to the considerable challenge posed by their high susceptibility to decay during storage. This spoilage issue is multifaceted, with fungal infections being a pivotal contributor to chestnut deterioration. Italian researchers reported for the first time the isolation of *Neofusicoccum parvum* from rotting chestnuts and identified it to be one of the main pathogens responsible for chestnut rot [6]. *Botryosphaeria dothidea* and *N. parvum* belong to the same family of *Botryosphaeriaceae,* and both were isolated from rotten chestnuts in our previous study [7]. The former has since been identified on a number of woody plants, including grapes, mangoes, olives, eucalypti, maples, and oaks, and is still expected to have a broad geographical distribution [8]. Researchers found that it also exists as an endophytic fungus in chestnut plant tissue [9]. As a plant pathogen, *B. dothidea* can cause ulcers on economic trees, colonize fruits, and induce fruit rot [10]. In our previous study, we isolated some pathogenic fungi, including *B. dothidea*, *N. parvum*, and *Fusarium proliferatum*, from decayed chestnut fruits [7]. Through further study, we found that *B. dothidea* has a strong infestation ability on chestnut, which may be an important causative factor for chestnut rot.

Perilla (*Perilla frutescens*) belongs to the genus *Perilla* of the family Perillaceae. *P. frutescens* comprises multiple varieties, including *P. frutescens* var. *purpurascens* (Hayata) H. W. Li and *P. frutescens* var. *crispa* (Thunb.) Hand.-Mazz [11]. Perilla, renowned for its abundance of phytochemicals, minerals, and vitamins, exerts a notable calming and anti-inflammatory influence on the body. Furthermore, it possesses preservative and sterilizing properties, making it an efficacious natural agent for maintaining the freshness and safety of other food items. Perilla leaves can be used to make dishes and to pickle kimchi. Various parts of the perilla, such as leaves and seeds, contain essential oils [12]. Perilla essential oil (PEO) has a good antifungal effect, antioxidant capacity, medicinal value, and preservation ability. Hu et al. [13] found that PEO treatment could suppress the growth of *Aspergillus flavus*, destroy the normal morphology and activities of cell walls and membranes, and cause defense dysfunction against stress response. Wang et al. [14] determined that the composite EO_x_-NNSC films were used to provide antioxidant and antifungal properties in food packaging. Perillaldehyde (PAE) is often considered the main active ingredient in PEO. PAE has a wide range of uses, including food seasoning, antifungal, antioxidant, and antitumor. It is gradually replacing synthetic fungicides and chemical drugs as a novel antifungal, antioxidant, and natural preservative for controlling infectious or postharvest diseases and decays in food crops and plants [15]. Many reports have been published on the wide application of PEO and PAE, especially their efficient antifungal activity in extending the shelf life of food [16]. Therefore, this study focused on the antifungal activity of PAE and PEO against the chestnut pathogen *B. dothidea*, assessed their antifungal mechanism, and provided further scientific basis for the use of essential oils in chestnut storage and even food preservation. In this study, a new drug was screened against *B. dothidea*, a broad-spectrum pathogen, which is not only useful for the control of chestnut rot caused by *B. dothidea* but also has the potential to be promoted for the prevention and control of *B. dothidea*-induced diseases on various cash crops.

## 2. Materials and Methods

### 2.1. Experimental Materials

Perilla leaves were purchased from Macheng, Hubei Province, China, and identified as *P. frutescens* var. *purpurascens* by Dr. Gao Zhou from the Hubei University of Technology. *B. dothidea* involved in this study was isolated from rotten chestnuts and identified by Prof. Qiang Cai from Wuhan University using morphological and other methods. The pathogen was numbered 20221022-6-1 and stored in a refrigerator at 4 °C using the slant low-temperature culture preservation method. Chestnut fruits were harvested in Yantianhe Town, Macheng City, Hubei Province.

The dried perilla leaves (250 g) were crushed and added to the Clevenger instrument together with 2500 mL of H_2_O. Steam distillation was used for 3 h to extract PEO. The extracted PEO was stored in a 4 °C refrigerator until next use. The PAE (GC) was obtained from Shanghai Aladdin Biochemical Technology Co., Ltd., Shanghai, China.

### 2.2. Characterization of PEO by GC/MS Analysis

The chemical characterization of PEO was accomplished utilizing a gas chromatography/mass spectrometry (GC/MS) analysis. A precise volume of 1 μL of the filtered sample was introduced into a GC/MS system (Agilent 7890/5975, Agilent Technologies, Santa Clara, CA, USA) and separated on an HP-5 ms column (30 m × 0.25 mm × 0.25 μm) (Agilent Technologies). Helium was used as the carrier gas set up at a flow of 1 mL/min. The GC oven temperature increased from 80 °C to 180 °C at a rate of 5 °C/min, and then increased to 260 °C at a 10 °C/min rate. The inlet, interface, ion source, and quadrupole temperatures were set at 250 °C, 250 °C, 230 °C, and 150 °C, respectively. MS data were acquired in the m/z range of 20–500 in full-scan mode with a solvent delay of 2.5 min. Essential oil compounds were identified by comparing their mass spectra with those provided by the National Institute of Standards and Technology (NIST17) database. The relative contents of each component were calculated by peak area normalization method. The identification was done following the method published by Liu [17].

### 2.3. Antifungal Assays In Vitro

#### 2.3.1. Agar Dilution Method

An agar dilution method was used to test the effects of PEO and PAE on the growth of *B. dothidea* [18]. Briefly, PEO or PAE was added to a PDA culture medium to its final concentrations of 0.125, 0.250, 0.500, and 1.000 μL/mL. The PDA medium (30 mL) containing PEO or PAE at different concentrations was evenly distributed into three petri dishes. After solidification, a *B. dothidea* mycelium block with a diameter of 5 mm was placed at the center. It was sealed with parafilm and placed in a constant-temperature (28 °C) incubator for 3 days. The radial expansion of *B. dothidea* mycelia under each treatment condition was quantified daily, employing a precision digital vernier caliper (MITUTOYO-ABS Digimatic Caliper CD-AX, Japan) for accurate measurements to the nearest millimeter. A minimum concentration of components that did not permit any visible growth was selected as minimum inhibitory concentration (MIC). The inhibition percentage of mycelial growth was calculated according to the following formula:

$$\%\;\mathrm{Inhibition}=\left(\frac{\mathrm{Control}\;\mathrm{mycelial}\;\mathrm{diameter}-\mathrm{Treatment}\;\mathrm{mycelial}\;\mathrm{diameter}\;}{\mathrm{Control}\;\mathrm{mycelial}\;\mathrm{diameter}\;}\right)\times 100$$

#### 2.3.2. Fumigation Method

The effects of PEO and PAE on the growth of *B. dothidea* were tested using a fumigation method [19]. Sterile filter paper containing different volumes of PEO and PAE was placed on the side of the Petri dish that did not contain culture medium, and a *B. dothidea* mycelium block with a diameter of 5 mm was inserted at the center of the culture dish containing PDA medium. The block was sealed with film to prevent PEO and PAE leakage and placed in a constant-temperature (28 °C) incubator for 3 days. Every 24 h, the diameter of hyphae was measured using a cross method, and a hyphal growth curve was drawn. The drug concentration in the air was calculated using the following formula:

C = V1/V2,

V1: Volume of PEO or PAE in filter paper; V2: Volume of Petri dishes.

#### 2.3.3. Fungal Biomass Determination

Fungal biomass was used to intuitively reflect fungal growth [20]. PEO or PAE dissolved in 0.1% Tween-20 was diluted in an Erlenmeyer flask containing 25 mL of PDB culture medium, so that the final drug concentrations were 0.063, 0.125, 0.250, 0.500, and 1.000 μL/mL. A piece of *B. dothidea* mycelium plug with a diameter of 5 mm was added to each Erlenmeyer flask, which was then placed in a shaker with a rotational speed of 200 rpm and a temperature of 28 °C. After 3 days, the mycelium was obtained by filtration, dried, and weighed.

#### 2.3.4. Scanning Electron Microscopy (SEM) Observation of the Effect of PEO and PAE on Mycelial Morphology

Hyphal morphology was observed in accordance with a previous method with slight modifications [21]. The *B. dothidea* mycelium plug was cultured on a shaking table in a PDB medium with 0.25 μL/mL of PEO or 0.25 μL/mL of PAE for 3 h. Then, all samples were incubated with 2% *v/v* pentanediol, placed in fixative at 4 °C for 24 h, and washed with 100 mM phosphate buffer (pH = 7.4). All samples were dehydrated with ethanol at different concentrations (30%, 50%, 70%, 90%, and 100%), critical point dried, gold-plated, and analyzed under a scanning electron microscope (S-3400, Hitachi, Tokyo, Japan) to observe the morphology of the mycelium.

### 2.4. Study on the Antifungal Mechanism of PEO and PAE against B. dothidea

#### 2.4.1. Effects of PEO and PAE on *B. dothidea* Cell Wall

The integrity of the cell wall is crucial to fungi and is one of the factors for testing antifungal drugs. In accordance with the method of Contreras et al. [22], the condition of the fungal cell wall after treatment with different concentrations of drugs was evaluated. *B. dothidea* mycelium suspension (100 μL) with a concentration of 1 mg/mL was added to an Erlenmeyer flask containing 25 mL of PDB culture medium, which was placed in a shaker with a temperature of 28 °C and a rotational speed of 200 rpm for 12 h. Different concentrations of PEO or PAE were added, and the final concentrations were 0.125, 0.250, and 0.500 μL/mL. The control group was not treated and cultured for 3 h. Subsequently, the hyphae were collected, added with 10 μL of calcofluor white (CFW) and 10 μL of KOH (10%) dropwise, and stained for 5 min in the dark. Excess dye was removed and observed with a laser confocal scanning microscope (Leica TCS SP8 CARS, Wetzlar, Germany).

#### 2.4.2. Effects of PEO and PAE on *B. dothidea* Cell Membrane

The status of fungal cell membranes after treatment with different concentrations of drugs was evaluated in accordance with the method of Contreras et al. [23]. The mycelial culture method was the same as that in Section 2.4.1. Different concentrations of PEO and PAE were added. The final concentrations were 0.125, 0.250, and 0.500 μL/mL. No treatment was performed on the control group, and the shaking culture was continued for 3 h. An appropriate amount of mycelium on the glass slide was collected, added with 0.5% Evans blue dye dropwise, stained for 5 min, and rinsed with PBS. The slide was placed under a microscope (Olympus CX23, Beijing, China) to observe the integrity of the *B. dothidea* cell membrane.

#### 2.4.3. Determination of Ergosterol Content

Ergosterol content was determined on the basis of a previous method with slight modifications [24]. A fungus block with a diameter of 5 mm was placed in an Erlenmeyer flask containing 25 mL of PDB and cultured in a shaker at a temperature of 28 °C and a rotational speed of 200 rpm for 48 h. Different concentrations of PEO and PAE were added to realize final concentrations of 0.25 and 0.50 μL/mL. The control group was not processed. The incubation was continued for 3 h. Then, the mixture was centrifuged at 8000 rpm and room temperature for 10 min, the supernatant was discarded, and 0.5 g of mycelium precipitate was collected from each group in a centrifuge tube, added with 5 mL of 25% potassium hydroxide ethanol solution, shaken and mixed for 10 min, and placed in a water bath at 85 °C for 4 h. A mixture of water and n-heptane (1:3, *v/v*) was added to the centrifuge tube, shaken and mixed for 10 min, and let stand for layering. The n-heptane layer was collected and kept at −20 °C for 24 h. A UV spectrophotometer was used to perform a full-wavelength scan between 230 and 300 nm. The ergosterol content was determined using the following formulas:

% Dehydroergosterol = (A230/518) × 100

% Ergosterol = (A282/290)/w × 100 − % Dehydroergosterol

In these equations, 518 and 290 are constants, and w is the mycelium wet weight.

#### 2.4.4. Effects of PEO and PAE on Cellular Content Leakage

The release of cellular contents was determined as described previously [25], with some modifications. The fungal culture and drug treatment were the same as those in Section 2.4.3. The mixture was centrifuged at 8000 rpm and room temperature for 10 min. The supernatant was collected, and the absorbance was measured at wavelengths of 260 and 280 nm. The leakage ratio according to the following formula:

$$\mathrm{Ratio}=\left(\frac{\mathrm{absorbance}\;\mathrm{of}\;\mathrm{treated}\;\mathrm{group}-\mathrm{absorbance}\;\mathrm{of}\;\mathrm{empty}\;\mathrm{plate}}{\mathrm{absorbance}\;\mathrm{of}\;\mathrm{untreated}\;\mathrm{group}-\mathrm{absorbance}\;\mathrm{of}\;\mathrm{empty}\;\mathrm{plate}}\right)$$

### 2.5. Antifungal Activity In Vivo

#### 2.5.1. Antifungal Effects of PEO and PAE on Chestnut Kernels

The experiment was performed in accordance with a previous protocol with slight modifications [26]. Fresh chestnut kernels were collected, washed with sterile water, cut into uniform cubes with a mass of 1 g using a blade, and placed in a Petri dish. All chestnut kernels were divided into control group, model group, low-dose group (0.5 μL/mL, MIC), medium-dose group (1.0 μL/mL, 2MIC), and high-dose group (2.5 μL/mL, 5MIC). Each group had three repetitions, in which each repetition consisted of five chestnut kernels. Appropriate amount of *B. dothidea* mycelium was weighed in a centrifuge tube, sterile water was added to make a final concentration of 1 mg/mL and prepared as a suspension of uniformly textured mycelium by grinding using a grinder. The suspension was then further dispersed using a vortex mixer. Except for those in the control group, chestnut kernels in other groups were inoculated with 20 μL of *B. dothidea* mycelial suspension at a concentration of 1 mg/mL. After sealing, they were placed in a constant-temperature (28 °C) incubator for observation. The criteria for chestnut rot are color change, unpleasant odor, obvious hyphae coverage on the surface, and reduced hardness. The percentage of disease incidence was calculated as follows:

$$\%\;\mathrm{Incident}\;\mathrm{rates}=\left(\frac{\mathrm{Number}\;\mathrm{of}\;\mathrm{rotted}\;\mathrm{fruits}}{\mathrm{Total}\;\mathrm{number}\;\mathrm{of}\;\mathrm{fruits}}\right)\times 100$$

#### 2.5.2. Preventative Effects of PEO and Perilla Leaves on Chestnut Fruit Rot

Fresh perilla leaves with 10% of the weight of healthy chestnut fruit were placed under the fresh chestnut fruit in a sealed container. Every 3 days was a cycle, and the rotting of the chestnut fruit was observed and recorded.

Completely browned healthy chestnut fruits were divided into PEO emulsion treatment groups and perilla leaf treatment groups. PEO emulsion consisted of 7.5% PEO, 5% sucrose fatty acid ester, and balanced distilled water. In PEO-treated groups, after the PEO emulsion was diluted, antifungal solutions with PEO concentrations of 1 μL/mL (minimum fungicidal concentration, MFC), 5 μL/mL (5MFC), and 15 μL/mL (15MFC) were obtained and sprayed on the surface of the chestnut fruit. Then the chestnut fruit was placed in a sealed container. In perilla leaf treatment groups, fresh perilla leaves were placed under the fresh chestnut fruit in a sealed container. The weight of PEO emulsion was 2% of the weight of chestnut fruit, and the weight of perilla leaves was 5% of the weight of chestnut fruit. The rot rate of chestnut fruits was observed in a 3-day cycle. The criteria for chestnut rot are color change, unpleasant odor, obvious hyphae coverage on the surface, and reduced hardness. The percentage of disease incidence was calculated as follows:

$$\%\;\mathrm{Incident}\;\mathrm{rates}=\left(\frac{\mathrm{Number}\;\mathrm{of}\;\mathrm{rotted}\;\mathrm{fruits}}{\mathrm{Total}\;\mathrm{number}\;\mathrm{of}\;\mathrm{fruits}}\right)\times 100$$

### 2.6. Statistical Analysis

All data were expressed as mean ± standard deviations. Each treatment included at least three replicates. The analysis software was SPSS27.0 (SPSS, Chicago, IL, USA), and one-way analysis of variance was conducted. Statistical significance was set to * *p* < 0.05, ** *p* < 0.01, and *** *p* < 0.001.

## 3. Results

### 3.1. GC/MS Analysis of Composition of PEO

The chemical constituents of PEO were comprehensively analyzed utilizing GC/MS, leading to the identification of 13 predominant components. These constituents collectively represent 98.23% of the total composition of PEO, providing a detailed insight into its chemical profile (Table 1). Among these, elemicin stands out as the most abundant, constituting 48.31% of the total composition. This is followed by (Z)-β-Farnesene at 11.43%, caryophyllene at 10.92%, myristicin at 10.85%, and perilla ketone at 7.42%.

### 3.2. In Vitro Antifungal Activity Results

#### 3.2.1. Effects of PEO and PAE on Mycelial Growth

**Figure 1:**
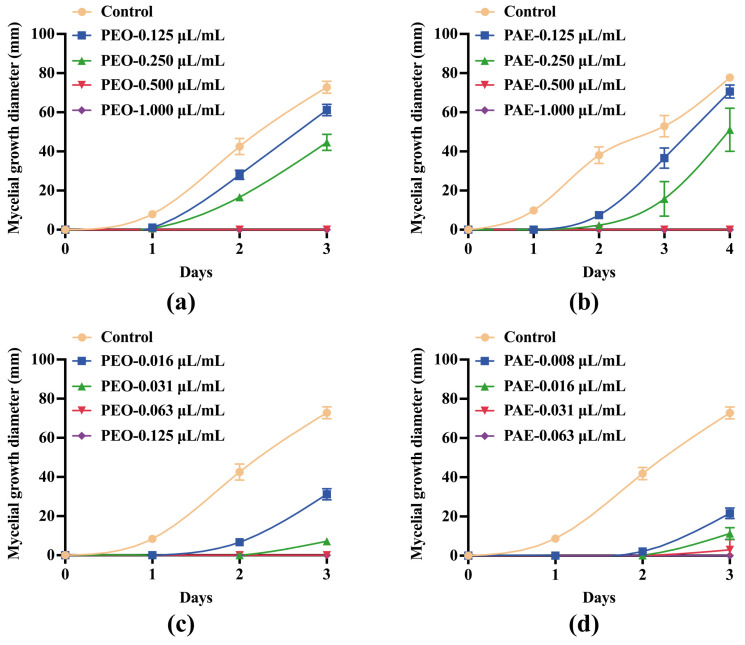
Effect of PEO and PAE on the growth of *B. dothidea*. (**a**,**b**) Growth of *B. dothidea* in a PDA medium containing PEO or PAE by plate dilution; (**c**,**d**) Growth of *B. dothidea* in a PDA medium containing PEO or PAE by fumigation.

The effects of different concentrations of PEO and PAE on the 3-day mycelial growth diameter of *B. dothidea* are shown in Figure 1. The 3-day growth inhibition rates of 0.25 μL/mL of PEO and PAE in *B. dothidea* were 36.23 ± 5.27% and 64.06 ± 15.30%, respectively. The growth of mycelia was completely inhibited at 0.5 μL/mL PEO treatment (Figure 1a,b). When the fumigation method was used, PEO and PAE showed excellent antifungal activity. When their concentrations reached 0.063 and 0.031 μL/mL, respectively, they could completely inhibit the growth of *B. dothidea* in 3 days (Figure 1c,d).

#### 3.2.2. Effects of PEO and PAE on Fungal Biomass

**Figure 2:**
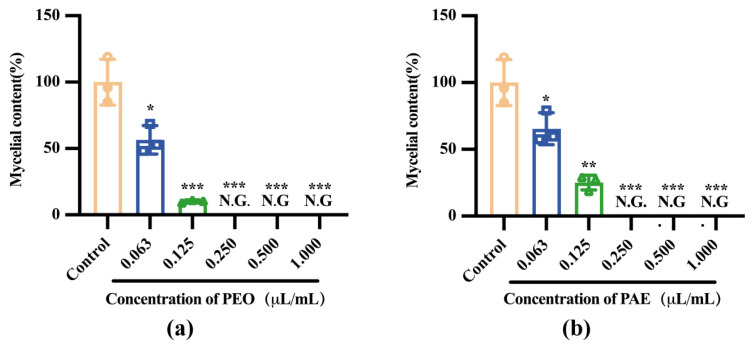
Effect of PEO and PAE on the growth of *B. dothidea*. (**a**) Relative mycelium content of *B. dothidea* after 3 days of growth in a PDB medium containing different concentrations of PEO; (**b**) Relative mycelium content of *B. dothidea* after 3 days of growth in a PDB medium containing different concentrations of PAE. N.G., No growth. Different color objects represent different treatment groups. * *p* < 0.05, ** *p* < 0.01, and *** *p* < 0.001 compared with the control.

As shown in Figure 2, PEO and PAE cultured in a PBD medium showed better antifungal activity than those in a PDA medium. When the concentration reached 0.125 μL/mL, the inhibitory rates of the two test drugs against *B. dothidea* were 89.69 ± 0.95% and 74.88% ± 5.55%, respectively. When the concentration reached 0.250 μL/mL, the inhibition of mycelial growth was completed.

#### 3.2.3. SEM Observation of the Effects of PEO and PAE on Fungal Morphology

**Figure 3:**
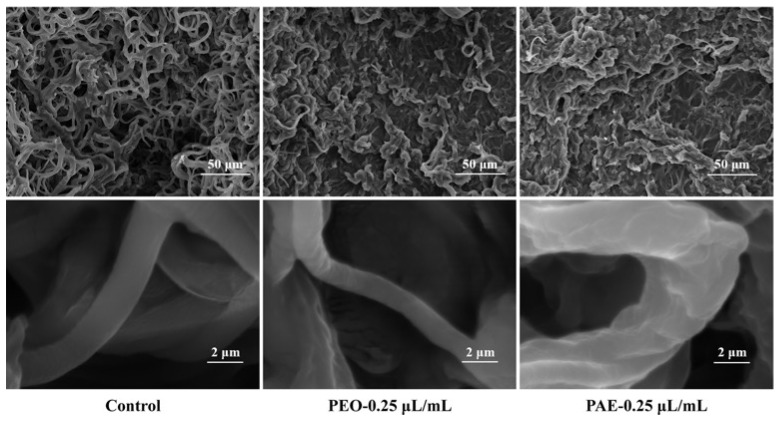
Effects of different concentrations of PEO and PAE on the fungal hypha morphology of *B. dothidea*.

SEM showed the changes in mycelial morphology caused by PEO and PAE (Figure 3). Compared with that in the control group, the mycelium in the PEO treatment group had many wrinkles on the surface and showed overall shrinkage, while the mycelium in the PAE treatment group had more wrinkles, severe collapse, and a disordered structure. The results showed that PEO and PAE at the lowest fungicidal concentration during PDB culture could cause morphological deformation or collapse of *B. dothidea*.

#### 3.2.4. Effects of PEO and PAE on *B. dothidea* Cell Walls

**Figure 4:**
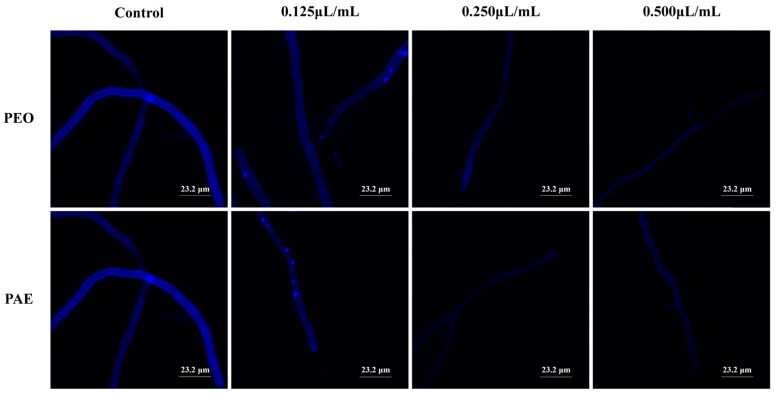
CLSM images of the mycelium treated with PEO or PAE at different concentrations after staining with CFW.

CFW was constantly used to analyze the alterations (e.g., localization and level of chitin) of fungal cell walls by monitoring the change in chitin, a major constituent of fungal cell walls [27]. As displayed in Figure 4, the control group mycelium showed complete blue fluorescence, depicting a normal distribution of chitin in the cell wall in the general mode. By contrast, the CLSM images of *B. dothidea* treated with different concentrations of PEO and PAE showed a faint blue color. The blue fluorescence became almost invisible as the drug concentration increased, indicating that the chitin content in the cytoplasm was greatly reduced at this time.

#### 3.2.5. Effects of PEO and PAE on *B. dothidea* Cell Membranes

**Figure 5:**
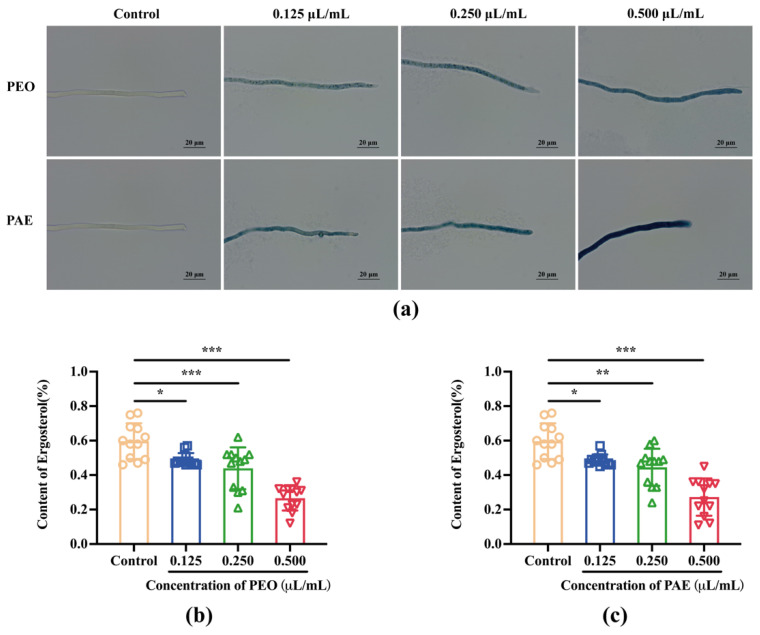
Effects of different concentrations of PEO and PAE on the fungal cell membranes of *B. dothidea*. (**a**) Mycelium image under a microscope after staining with Evans blue; (**b**,**c**): Ergosterol content in the mycelium cell membrane; * *p* < 0.05, ** *p* < 0.01, and *** *p* < 0.001 compared with the control. Bar = 20 μm.

The cells were stained with Evans blue staining to investigate if a disruption of cell membrane integrity occurred under PEO or PAE exposure. As indicated in Figure 5a, compared with the control group, when fungi were treated with PEO or PAE at a concentration of 0.125 μL/mL, most fungal cells were stained blue, indicating that the fungal cell membrane was damaged after 3 h of treatment with PEO or PAE. As the concentration increased, the blue color darkened, implying that the high concentration of the drug caused more serious damage to the fungal cell membrane.

Ergosterol is an important component of fungal cell membranes and determines the fluidity, permeability, and activity of membrane-associated proteins. Defects in sterol biosynthesis can lead to pleiotropic defects that limit cell proliferation and stress adaptation [28]. As indicated in Figure 5b,c, compared with that of the control group, the ergosterol content of fungal cells treated with PEO or PAE was significantly reduced in a dose-dependent manner. That is, PEO and PAE destroyed the integrity of the cell membrane by inhibiting the synthesis of ergosterol in fungal cells, achieving an antifungal effect.

#### 3.2.6. Nucleic Acids and Proteins Released from Damaged Membranes

**Figure 6:**
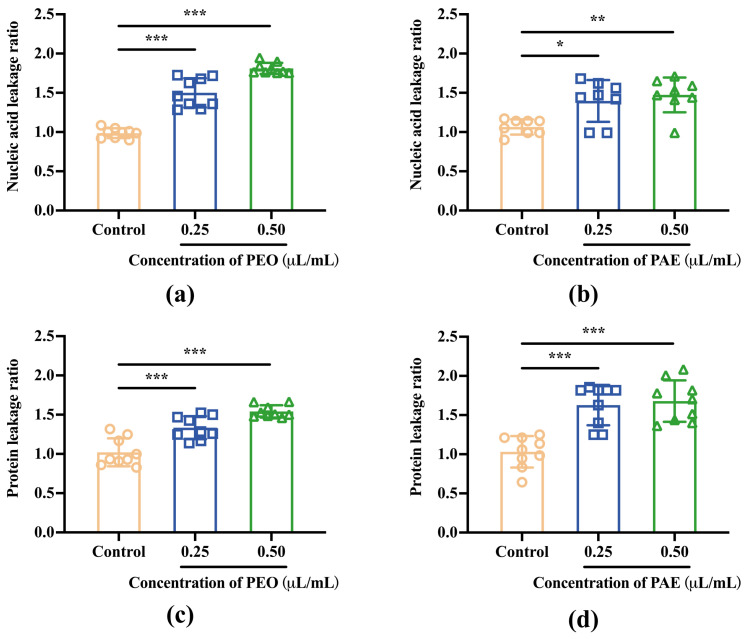
Effect of PEO and PAE on the leakage of the cellular contents of *B. dothidea*. (**a**,**b**): Detection of nucleic acid leakage at 260 nm; (**c**,**d**): Detection of protein leakage at 280 nm; * *p* < 0.05, ** *p* < 0.01, and *** *p* < 0.001 compared with the control group.

The contents of nucleic acids (OD260 nm) and proteins (OD280 nm) in the medium were measured for treated *B. dothidea* cells to further study the disruption of *B. dothidea* cell membrane. As displayed in Figure 6, compared with those of the control group, the nucleic acid and protein leakage rates of *B. dothidea* cells treated with PEO or PAE increased significantly with the increase in drug concentration. Hence, these antifungal drugs destroyed fungal cell membranes, achieving antifungal purposes.

### 3.3. Antifungal Activity In Vivo

#### 3.3.1. Antifungal Effects of PEO and PAE on Chestnut Kernel Pieces

**Figure 7:**
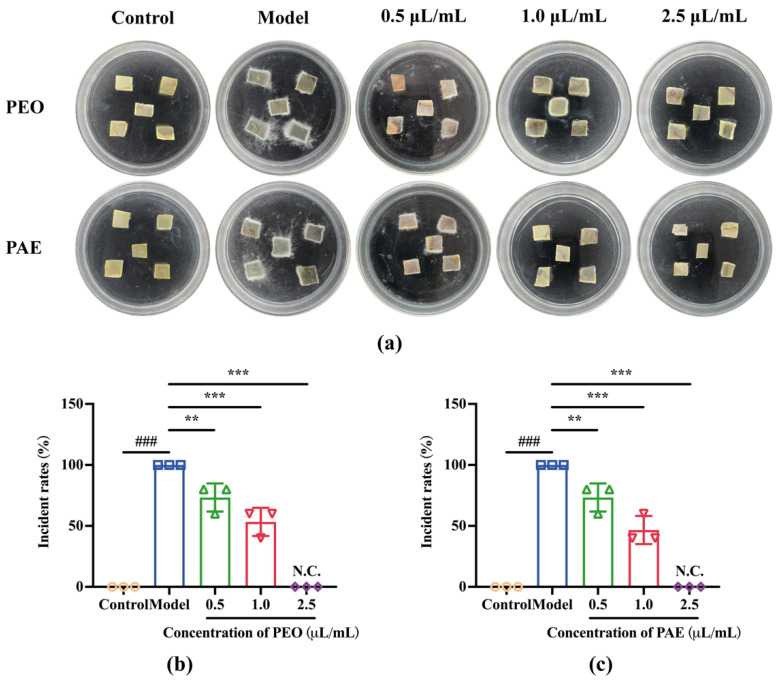
In vivo antifungal activity of PEO and PAE against the growth of *B. dothide*a. (**a**) Photos of fungal growth in chestnut kernels during the 7-day culture period; (**b**,**c**) Incidence of chestnut kernels treated with different concentrations of PEO or PAE. “Incident rates” represent the rotting rates of *B. dothidea*-infested chestnut kernel pieces in each treatment group. ### *p* < 0.001 compared with the model; ** *p* < 0.01, and *** *p* < 0.001 compared with the control.

As a recalcitrant seed, the structure of the chestnut is mainly composed of cotyledons, and the tissue mass of the chestnut has strong resistance and can survive under suitable temperatures and humidity. Chestnut kernel pieces show strong resilience and are able to survive under appropriate temperature and humidity conditions to maintain their structural integrity, while the rotten chestnut will soften and lose its due strength, change color, and grow hyphae, which can be seen clearly by the naked eye. Referring to our previous experimental methods [26], we used the aseptic chestnut tissue blocks after screening to build a fungal infection model to evaluate the control efficacy of our test drugs. Visual evidence was used to demonstrate the impact of PEO or PAE on the incidence of chestnut kernels inoculated with *B. dothidea*. As displayed in Figure 7, after 7 days, the chestnut kernels in the control group were still intact, whereas the chestnut kernels in the model group were covered with fungal hyphae. PEO or PAE at 0.5 μL/mL (MIC) and 1.0 μL/mL (2MIC) could effectively reduce the incidence of chestnut kernels, and the hyphal area on chestnut kernels was significantly reduced. PEO or PAE at 2.5 μL/mL (5MIC) could completely inhibit fungal activity, and no disease was found in chestnut kernels in this treatment group.

#### 3.3.2. Preventative Effects of PEO and Perilla Leaves on Intact Chestnut Fruit Rot

**Figure 8:**
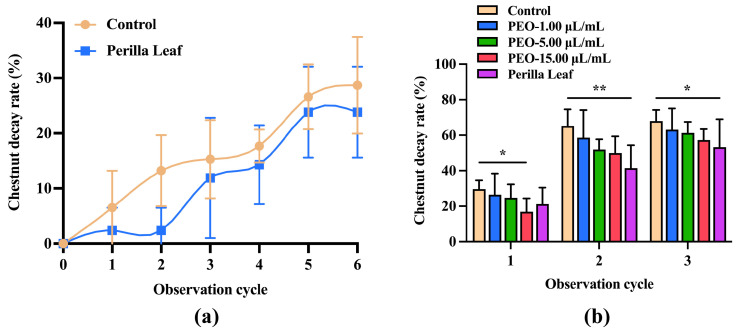
Decay rate of chestnut fruits after different treatments. (**a**) Rotting rate of unbrowned chestnut fruits after paving with perilla leaves; (**b**) Rotting rate of browned chestnut fruits treated with PEO or perilla leaves. * *p* < 0.05 and ** *p* < 0.01 compared with the control.

The above experiments showed the inhibitory effect of PEO on *B. dothidea* in vitro and the protective effect of fungal infections of the chestnut kernel in vivo. Furthermore, the effects of PEO and perilla leaves on the decay rate of intact chestnut fruits were explored. As shown in Figure 8a, compared with those in the control group, chestnut fruits in the perilla leaf bedding group exhibited a trend of reduced rot, which was also reflected in browned chestnut fruits. From Figure 8b, unlike those in the control group, chestnut fruits in the PEO and perilla leaf treatment groups showed varying degrees of reduced decay. In particular, the rot rate of chestnut fruits treated with PEO emulsion showed a downward trend within three observation periods (9 days) and was dose-dependent. Thus, PEO emulsion could effectively inhibit the activity of *B. dothidea* in chestnut fruits. Perilla leaves still had a significant inhibitory effect on the decay rate of browned chestnut fruits after three cycles. PEO, as the active ingredient of perilla leaves, exerted a certain inhibitory effect on the spoilage rate of chestnuts during storage and could extend the storage period of chestnuts.

## 4. Discussion

The rot problem of chestnuts during postharvest storage is caused by multiple factors, including fungal infection, and *B. dothidea* is one of the fungi with strong infection ability. *B. dothidea* and other species in the Botryosphaeriaceae have been recognized primarily as endophytes that infect healthy tissue of woody plants and remain dormant until the onset of stress conditions [29]. Climate change is expected to increase the stress on many plant communities, including trees in natural woody ecosystems, managed forests, and agriculture [30]. Consequently, the potential impact of Botryosphaeriaceae in general, but specifically *B. dothidea*, which is a widespread pathogen already present as an endophyte in numerous plant communities in various parts of the world, might be exacerbated [31]. In this study, we identified a new natural active ingredient that can control this fungus. PEO has been proven to have good inhibitory effects on *Staphylococcus aureus*, *Escherichia coli*, and *Shigella dysenteriae* [32]. The food properties of chestnut foreshadow the need for a highly effective and eco-friendly fungicide with no threat to human health, which coincides with the natural compound properties of PEO. As a widely distributed medicinal plant, *P. frutescens* contains multiple varieties, such as *P. frutescens* var. *frutescens*, *P. frutescens* var. *purpurascens*, *P. frutescens* var. *auriculatodentata*, *P. frutescens* var. *auriculato-dentata*, and *P. frutescens* var. *crispa*. *P. frutescens,* which are also divided into many chemical types based on the differences in essential oil. The main components in the *P. frutescens* var. *purpurascens* essential oil we used in this study were identified to be elemicin (48.31%) and myristicin (10.85%), which conform to the characteristics of the PP-em type of *P. frutescens* [33]. As the main component of PEO, elemicin, caryophyllene, and myristicin have been proven to have antifungal activity [18,34,35,36]. Elemicin exhibited significant efficacy for the preservation of food commodities because of its strong antifungal, antiaflatoxigentic, and antioxidant activities. A study revealed that elemicin significantly inhibited the maximum ergosterol biosynthesis at the lower doses than MIC [37]. A study had also found that the essential oil of nutmeg containing 21% of myristicin caused a decrease in the ergosterol content of the fungus’s plasma membrane, which caused cellular ion leakage [38]. The mechanism of action of caryophyllene is focused on the cellular membrane [39]. Moo et al. [40] reported that it alters the membrane permeability as revealed by Zeta-potential measurements. This membrane permeability leads to extravasation of cellular content and death. In this research, PEO and PAE were found to have similar inhibitory activities against *B. dothidea* by agar dilution, with MICs of 0.500 μL/mL and MFCs of 1.000 μL/mL. The inhibitory activities of both might have been affected by the way the fungus was treated. This conclusion was reached through fumigation, in which the MICs of PEO and PAE against *B. dothidea* were 0.063 and 0.031 μL/mL, respectively. The inhibitory activity of *B. dothidea* administered by fumigation was much stronger than that by direct contact with the PDA medium; this conclusion is the same as that of a previous study [41]. In the in vivo inhibition assay, the inhibitory activities of PEO and PAE were reduced compared with those in vitro, and the growth of *B. dothidea* was completely inhibited at a concentration of 2.5 μL/mL (5 MIC). This result is similar to the conclusion of a study using essential oils to resist *Botrytis cinerea* in tomatoes. That is, the MIC of essential oils in tomatoes in vivo and in vitro was nearly 10 times different in the vapor contact assay [42].

No reports existed on the antifungal mechanism of PEO and PAE against *B. dothidea*. In some previous studies, the most widely known and investigated antimicrobial mechanisms of antimicrobial agents have focused on fungal targets and physiological processes, such as disruption of fungal cell membranes and cell walls, inhibition of protein synthesis, and oxidative damage [43]. SEM observation showed that after PEO or PAE treatment, the mycelium structure collapsed, and the surface was covered with wrinkles, which, to a certain extent, indicated the loss of fungal cell cytoplasm and damage to organelles. Similar observations were made in a previous study by Jiang et al. [44]. The cell wall is an essential component in the homeostasis of fungal cells [45]. It has a dual interaction process with the surrounding environment, which either negatively or positively impacts fungal cell survival [46]. This research showed that the chitin content in the fungal cell wall was reduced after PEO or PAE treatment, indicating that the cell wall is one of the targets of antifungal drugs, which is the same as the findings of previous studies [22,27,45].

Evans blue was used to test the integrity of the fungal cell plasma membrane. The results showed that the permeability of the plasma membrane was affected after treatment with antifungal drugs. The plasma membrane functions as a physical border between the extracellular and cytoplasmic environments that contribute to the interaction between host plants and pathogenic fungi. As a specific sterol constituent in the cell membrane, ergosterol plays a significant role in fungal development [47]. By measuring the ergosterol content, this study found that the content in the drug treatment group decreased. The levels of nucleic acid and protein leakage were also measured, and plasma membrane disruption was found to be used by PEO and PAE for antifungal purposes. This finding is consistent with the conclusions of some previous studies [23,28,37,47].

In previous studies, antifungal drugs were often applied directly to food fruits to observe the deterioration of the fruits [48,49,50]. Similarly, after we added PEO or PAE to chestnut kernels, the mycelial growth on chestnut kernels was inhibited to varying degrees, indicating that PEO and PAE could effectively prevent the infection of chestnut kernels by *B. dothidea*. We also explored the effect of perilla leaves, as a botanical source of PEO, on chestnut fruits. From the 6th day to the 9th day, the rot rate of chestnut fruits on perilla leaves was significantly reduced, which might be due to the volatile antifungal components such as elemicin in perilla leaves [18].

The sensory evaluation of chestnuts is a critical aspect in assessing the quality of chestnut-based foods, significantly impacting their palatability and flavor. Kan et al. [51] found that all texture characteristics of boiled chestnut showed a significant correlation with total starch content. The moisture content demonstrated a significant positive correlation with taste, color, sweetness, texture, and aroma, indicating that moisture is closely related to sensory appearance, texture, and overall acceptability of the cooked chestnut. Furthermore, there was a positive correlation between water-soluble protein and the color and aroma of the cooked chestnut kernel [52]. In this study, the decay rate of PEO-treated chestnut fruits showed a decreasing trend, and at the same time, normal chestnut fruits did not exhibit dehydration within the drug action cycle. Our treatment primarily targets the outer shell of the chestnut, with minimal impact on the chestnut kernel itself. This is positively significant for the palatability and flavor of chestnuts. Further research can involve measuring the total starch content, total reducing sugar content, and water-soluble protein to explore the effect of essential oil treatment on the flavor of the chestnut kernel.

## 5. Conclusions

*B. dothidea*, as one of the chestnut pathogens, was investigated for the antifungal effect of PEO and PAE on it in chestnuts in vitro and in vivo, and the mechanism of inhibition was explored to some extent. The results showed that PEO with elemicin as the main component and PAE had strong inhibitory effects on the growth of *B. dothidea*, and their inhibitory effects may be realized by disrupting the cell wall and plasma membrane of *B. dothidea*. Fumigation of chestnuts during the post-harvest period with volatile compounds is an economical and effective way to prevent chestnut rot. These results suggest that PEO is a nontoxic and environment-friendly plant-derived fungicide that, together with PAE, can be used to control postharvest storage rot of chestnuts caused by *B. dothidea*. Therefore, a new drug was screened against *B. dothidea*, a broad-spectrum pathogen, which is not only useful for the control of chestnut rot caused by *B. dothidea*, but also has the potential to be promoted for the prevention and control of *B. dothidea*-induced diseases on various cash crops. However, extensive research is required to overcome the challenges regarding sensory aspects and obtain safer dosage limits.

## 6. Patents

CN116508556A, CN116473069A.

## Figures and Tables

**Table 1:** Chemical composition and content of PEO.

Peak No.	Retention Time (min)	Component	Molecular Weight	Relative Content (%)
1	9.670	Perilla ketone	166.2170	7.42
2	10.461	1-(furan-2-yl)-4-Methylpentan-1-one	166.2170	0.24
3	11.187	Isoegomaketone	164.2011	3.93
4	12.961	β-Elemene	204.3511	0.20
5	13.698	Caryophyllene	204.3511	10.92
6	14.478	(Z)-β-Farnesene	204.3511	11.43
7	15.140	Germacrene D	204.3511	0.95
8	15.407	(Z, E)-α-Farnesene	204.3511	0.46
9	16.027	δ-Cadinene	204.3511	0.14
10	16.155	Myristicin	192.2100	10.85
11	16.849	Elemicin	208.2500	48.31
12	17.266	Caryophyllene oxide	220.3505	3.02
13	17.715	Benzene,1,2,3-trimethoxy-5-(1E)-1-propen-1-yl-	208.2500	0.36
All				98.23

## Data Availability

The original contributions presented in the study are included in the article, further inquiries can be directed to the corresponding author.
